# Fire Inside the Cavity of a Non-flammable Facade: Step-by-Step Development of Multiphysics Computer Simulations

**DOI:** 10.1007/s10694-024-01680-z

**Published:** 2024-12-24

**Authors:** Benjamin Khoo, Wolfram Jahn, Matthew Bonner, Panagiotis Kotsovinos, Guillermo Rein

**Affiliations:** 1 Department of Mechanical Engineering, Imperial College London, London, UK; 2 Arup, Manchester, UK; 3 Department of Mechanical and Metallurgical Engineering, Centro Nacional de Excelencia para la Industria de la Madera, Pontificia Universidad Católica de Chile, Santiago, Chile

**Keywords:** Flame, CFD, Flow, Turbulence, Buoyancy, Combustion

## Abstract

The cavities in a building facade can significantly increase the fire hazard, acting as pathways and accelerators for the vertical spread of flames and smoke, even in non-combustible facades. Ensuring fire safety during facade design requires a thorough understanding of how cavity geometry influences fire dynamics. However, established theories for this phenomenon are lacking. Therefore, in this study, we use the computational fluid dynamics code FireFOAM to develop step-by-step multiphysics simulations incorporating fluid mechanics, heat transfer, buoyancy, and combustion phenomena to investigate the non-linear behaviour in narrow vertical cavities. Four scenarios of increasing complexity are modelled and validated against experimental data from the literature. The simulations predict flow velocities and convective heat fluxes within 20% error and buoyancy-driven flow, radiative heat flux, and flame height predictions within 30% error across a range of cavity widths. The study also highlights the limitations of the models, offering insights for future refinement. The results demonstrate that computer simulations can reliably be used to study critical phenomena of cavity fires and, with future improvements, predict fire behaviour across various facade designs and conditions.

**Supplementary Information:**

The online version contains supplementary material available at 10.1007/s10694-024-01680-z.

## Introduction

A facade is one of the most complicated systems of high-rise buildings that can cost up to 25% of the total budget [1]. This is due to its multiple objectives, such as but not limited to aesthetics, minimising water ingress, and maximising sound insulation, that facades must achieve to provide a high-performing, safe, and comfortable environment [2].

According to Bonner et al. [3, 4], the frequency of facade fires has quickly increased in recent years to an annual average of 4.8 fires worldwide in 2020. In addition to the increased frequency, recent facade fires are often more severe as modern facades become more flammable [5].

One typical feature of modern facade systems is the presence of cavities in rainscreen facades. Cavities allow air to ventilate and remove moisture from insulation. This increases the facade’s lifespan by reducing water damage and degradation [2]. With regard to fire safety, the presence of a facade cavity may contribute to vertical fire and smoke spreading to other floors [6, 7]. As the flame enters the cavity, the temperature difference between the fire inside and air outside the cavity increases the upward buoyant flow, enhancing flame extension and spread [8]. This process, known as the chimney effect, can extend the flame height by up to ten times [9] and greatly increase re-radiation heat transfer to the walls of insulation and cladding [10]. Therefore, the presence of a cavity can increase fire hazards, especially when combustible materials are used in the cladding or the insulation [4]. To address this fire hazard, fire engineers routinely design cavity barriers into facade systems. Cavity barriers are construction within a cavity that limit smoke or flame entering the cavity, or restrict movement or flame within the cavity [11]. However, it is important to note that depending on the material of the cladding, the cavity barriers could be rendered ineffective if the cladding degrades quickly [12–14]. Despite the considerable effect of the cavity on fire hazards, there are currently no established theories that study their impact [4].

One of the first studies on facade cavities was performed by Lie in 1972 [15]. Lie used a channel 2.34 m tall and 0.91 m wide with an inert wall and one side made of polymer insulation. At the bottom of the channel, a furnace was used as a heat source. Lie found that the flame spread within the cavity depends on the flammability of the insulation. Further work on the effect of cavity width by Choi and Talyor [16, 17] found a critical width for continuous flame spread over the insulation. They found that when the cavity width is too narrow, the airflow feeding the flames becomes insufficient, which suffocates combustion and hinders heat transfer, reducing the pyrolysis rate and the fire power [17].

Several researchers investigated the heat transfer of cavity fires [7, 18–21]. These studies found that heat transfer by convection and radiation to cladding increases as cavity width reduces due to changes in airflow. Foley et al. [20] investigated the effect of cavity width with two parallel inert walls with open sides. They found that heat transfer to the wall becomes sensitive to cavity width if the base is sealed due to high air entrainment from the sides. The air entrainment from the sides pushed the flames towards the centre, resulting in higher heat flux at the centre of the wall [20]. They also found that the position of the burner between the walls affected the heat transfer, where heat flux does not simply reduce when the burner is placed further laterally from the wall. A recent investigation by Guillaume et al. [22] using an intermediate-scale experiment found that combustible claddings play a significant role in facade fires. They found that heat flux on the cavity wall changes drastically depending on the combustibility of the cladding, and cavity barrier effectiveness reduces when the cladding degrades or burns away.

More recently, Livkiss et al. [23] used two inert parallel walls with open sides to investigate the effect of narrow cavity width on flame height and heat flux on a non-combustible panel. In this study, the burner power per base length was varied, and the cavity width, W, was varied between 20 and 100 mm. The experiments found the flame heights can be constant or increased by up to 2.2 times compared to open fire depending on the burner power and cavity width. The study also showed that heat flux on the cavity wall increases as the cavity width decreases.

While some studies [7, 18–20, 23] suggest that reducing cavity width would increase incident heat flux on the wall, a narrow cavity does not always result in a fire with the highest heat release rate (HRR). A study by Jamison et al. found that a facade system with combustible insulation produces a fire with a larger HRR when the cavity is wider [24]. In this study, Jamison et al. experimented with a parallel panel with polymer insulation installed on one panel with different cavity widths. The results suggest that a wider cavity results in a higher HRR due to increased air entrainment. Bonner et al. [4] have also put forward a hypothesis that suggests as facade cavity width increases, more flames could enter the cavity due to the chimney effect, resulting in a higher HRR within the facade cavity.

These experimental studies suggest that the facade cavity fire problem is complex, and fire dynamics within the facade cavity are non-linear. Therefore, more experiments are needed to improve our understanding of how cavity width affects fire dynamics. However, one of the main challenges in conducting facade experiments is the complexity and the time needed to set up the rig, which may have contributed to the scarcity of available data on large-scale facade fires. One complementary way to study the effect of facade cavity width is to use numerical methods to augment the experiments. Numerical studies can be used to investigate different parameters without substantial additional costs. Several studies have developed models to study the fire dynamics within a cavity. One of the first models to study cavity-like fire was a two-dimensional model developed by Ingasson to study rack storage fire [25]. In this study, the model predicted flow velocities and temperatures well but not flame height. A later study by Yan et al. [26] developed and validated a 3D RANS model for a cavity fire. The model was able to reproduce the effect of the cavity where heat flux on the wall increases with cavity width qualitatively. However, the model underpredicted heat fluxes at the higher locations due to the poorly predicted buoyancy. Smoke movement within cavities has also been studied by various researchers. [27, 28] with a focus on double-skin facades with cavity widths varying from 0.5 to 2.0 m. However, these models were not validated against experimental results and were only used qualitatively. More recently, Drean et al. used LES CFD model to complement previous experimental findings on an intermediate-scale facade cavity fire [29]. The model shows good overall agreements, but it was developed to study flammable material and thermal degradation was modelled by assigning a specific burning rate. Lastly, Livkiss et al. compared LES CFD model to their experiments [30]. The model shows predictions of flame height and velocity within 20% of the experimental measurements. Livkiss et al. also offer a guide for simulating facade cavity fire. They suggested that at least 10 grid cells are required to obtain good predictions within a facade cavity.

**Figure 1:**
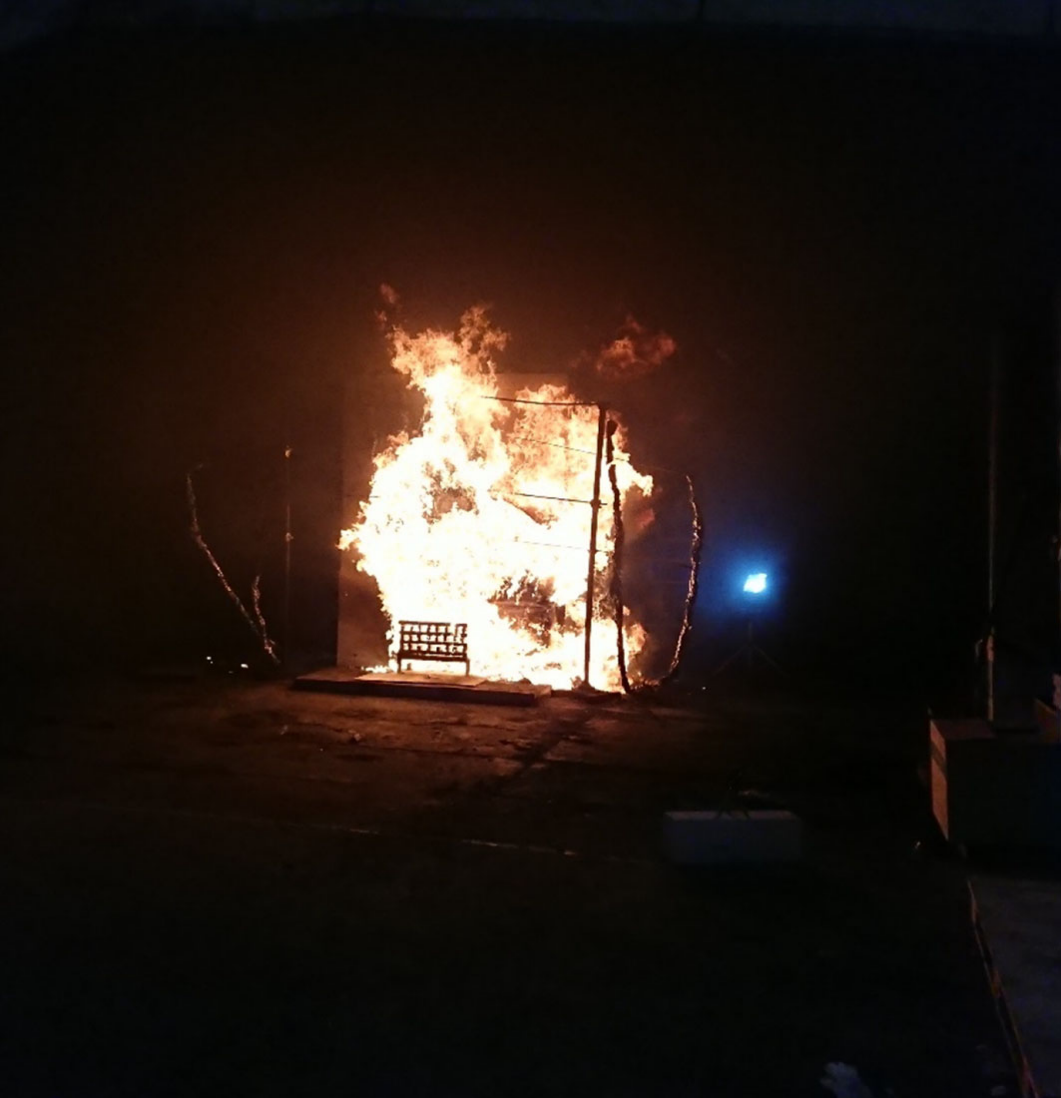
Example of facade testing to study the flammability of the facade system

The development of these models is essential in understanding cavity fire. However, as cavity fire is a multiphysics phenomenon, all relevant physics must be validated for a narrow cavity scenario to ensure the model is robust for extrapolation for different facade configurations. Current cavity fire models validate these physics all at once, creating a model that might include significant compensation effects. The compensation effect is the concept where results could be obtained by simultaneously varying two or more parameters [31, 32]. This creates a perception that the model is validated when a combination of two or more wrongly predicted physics could result in an acceptable prediction. Therefore, validating all the submodels at once may give false confidence that the model can accurately predict the multiple physics. In this paper, we separated the phenomena of importance in a cavity fire and validated each physical phenomenon one at a time with FireFOAM, a fire-specific model built on the OpenFOAM libraries. In the current study, the effect of pyrolysis in a narrow cavity is not considered, and a forthcoming paper will address pyrolysis in a narrow cavity (Figure 1).

## Methodology

**Figure 2:**
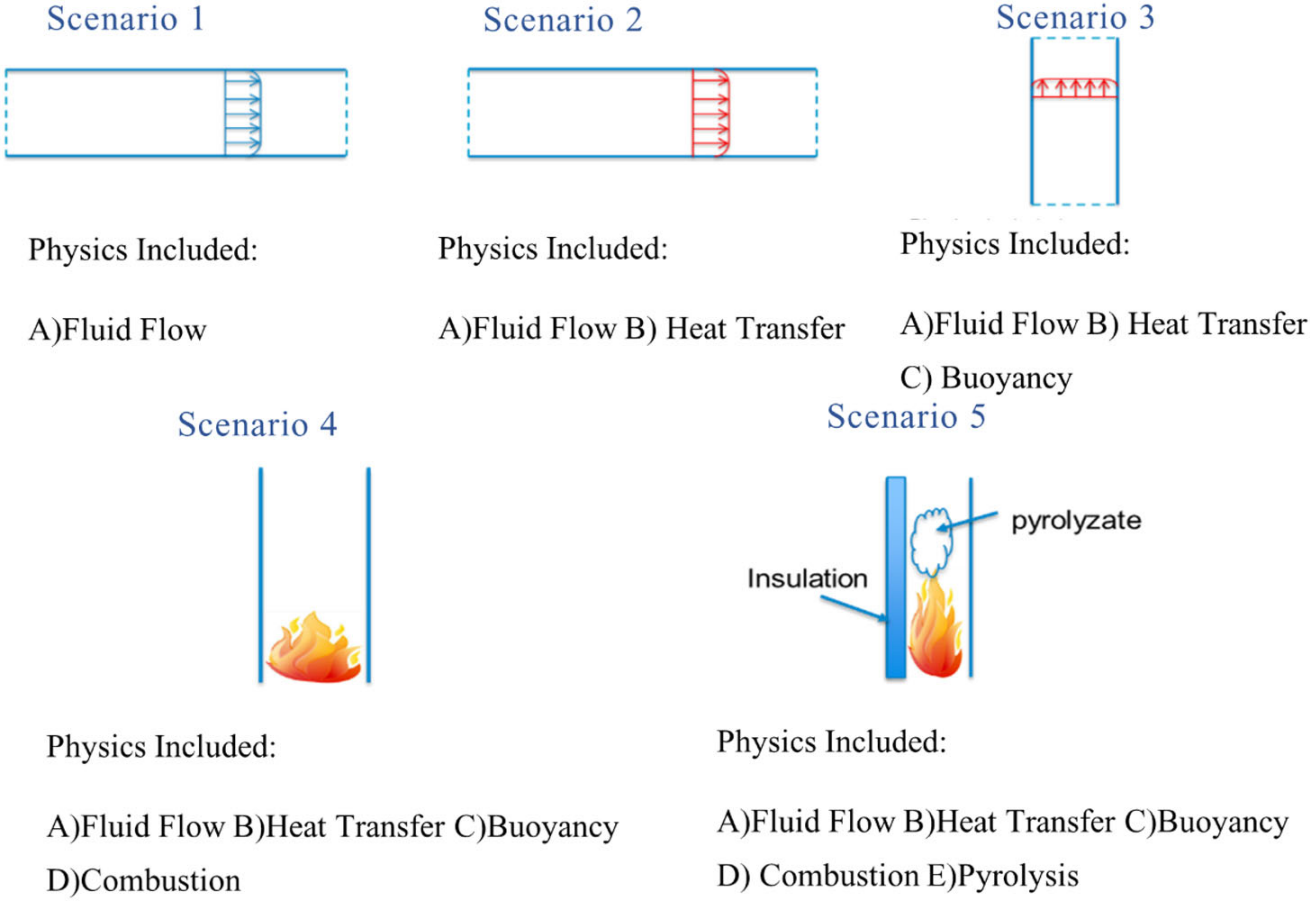
The different physics involved in a cavity fire and the corresponding scenario to validate the model’s physics. Each physics interacts with the other in the facade cavity to create a non-linear fire behaviour

The most important physical phenomena in cavity fire are fluid flow, heat transfer, buoyancy, pyrolysis and combustion. We split these into five different scenarios with increasing complexity, as shown in Figure 2. The rationale behind this approach is to limit the compensation effects of the model when simulating the strongly coupled cavity fire scenario. To achieve this, the model parameters are fixed and carried forward to the next scenario as each scenario is validated. Various researchers have already extensively studied the first three scenarios [33–45]. The approach to simulate these scenarios again as part of this work is to establish confidence and to find the different model parameters.

Only Scenarios 1 to 4 are presented here. The integration of pyrolysis chemistry into the CFD model corresponds to a major leap in terms of complexity. Scenario 5 which validates pyrolysis chemistry, is presented in future papers. The experimental data used to validate these scenarios are found in the literature [23, 33, 35, 37]. Note that while the validation cases are not in the range of actual fire conditions, the validation of these scenarios represents the first step in developing a robust model that can be used to study various cavity fire scenarios.

## Experimental Setup

**Figure 3:**
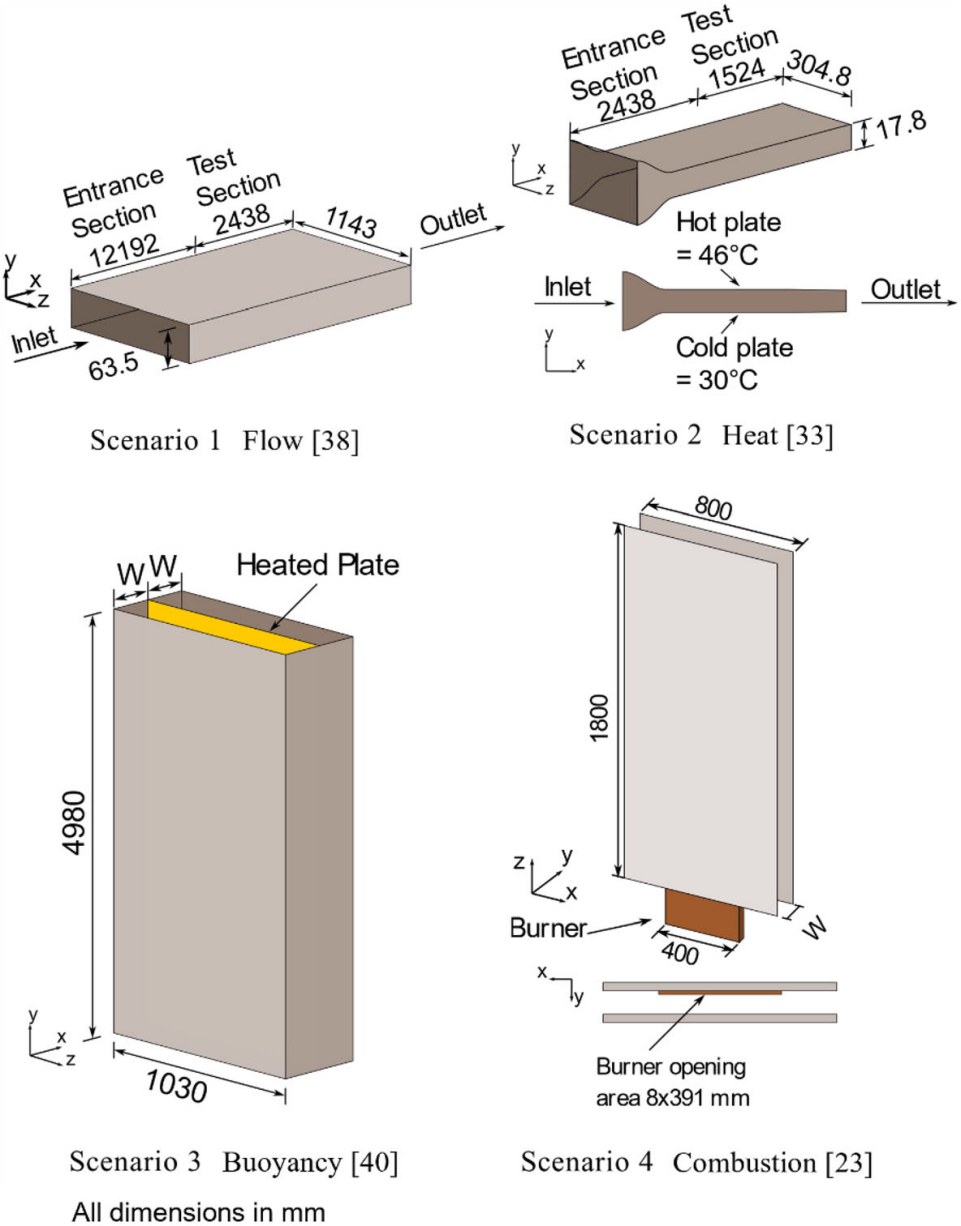
Simplified schematics of Scenarios 1 to 4. Each scenario represents one of the multiple important phenomena the model intends to validate. The drawings are not to scale

The experimental setup of each scenario will only be discussed in brief, and the readers are referred to the original research for more details on Scenario 1 [35], Scenario 2 [33], Scenario 3 [37], and Scenario 4 [23]. Simplified schematics of the experiments are shown in Figure 3.

For Scenario 1 Flow, the experiment consists of a narrow horizontal channel that is 14,630 mm long, 1143 mm wide and has a cavity width of 60.35 m. The channel is enclosed on the sides such that no inflow or outflow is induced laterally. The airflow through the channel has a bulk velocity of 5.77 m s^−1^ (Re = 13,8000). The velocity profile was measured using an anemometer in the test section at x = 12,192 mm.

Scenario 2 Heat setup is similar to that of Scenario 1, with a shorter and smaller setup with its length, width and cavity width are 3962 mm, 304.8 mm, and 17.8 mm, respectively. The air is blown through the cavity at a mean velocity of 4.66 m s^−1^ (Re = 9370). The ambient air temperature is 37°C, and the bottom and the top plate temperature are 30°C and 46°C, respectively. An anemometer and thermometer are used to capture the velocity and temperature profiles of the flow at x = 2438 mm.

In Scenario 3 Buoyancy, a parallel channel of 4.98 m height and 1.03 m width was constructed with a vertical plate with a uniform heat generation plate placed between two polycarbonate plates. The experimental result found that the temperature gradient near the polycarbonate plates is small, and therefore the panel is assumed to be adiabatic. Similarly, the channels are enclosed to prevent lateral airflow. The distance between the heated plate and adiabatic plates is identical and separated by distance W, where W = 50 mm, 100 mm, and 200 mm. The generated wall heat flux investigated in the paper was 104 W m^−2^ and 208 W m^−2^. Velocities were measured along the cavity at the height of 0.82 m, 2.665 m and 3.865 m using LDV and temperatures were measured using a thermocouple along the heated wall.

Scenario 4 Combustion setup consists of two parallel non-combustible vertical walls with a height of 1.8 m and width of 0.8 m with the sides. A propane gas burner with an opening of 8 mm × 391 mm burner was placed at the centre of the cavity next to a vertical plate. This plate is hereby referred to as “near wall”, whereas the other is referred to as the “far wall”. The cavity width and burner heat release rate (HRR) studied in this experiment vary from 20 mm to 100 mm and 6.5 kW to 15.8 kW, respectively. Measurements in this experiment consist of exit flow velocities at the top measured using bi-directional probes and heat flux.

The experiments measured three parameters of interest: flame height, heat flux and flow velocities which are essential in validating the robustness of the model. The validation of Scenario 4 Combustion represents the main step in developing a robust simulation of cavity fires.

## Numerical Modelling

The simulations were performed using FireFoam-dev [46], a solver based on the open-source framework, OpenFOAM. OpenFOAM provides numerical routines for solving partial differential equations by discretising them using the Finite Volume technique on both structured and unstructured meshes [47]. FireFoam is an unsteady solver for buoyancy-driven turbulent reacting flows. It is a LES-based solver that solves the Navier–Stokes equation using a fully compressible flow formulation. The FireFOAM code assumes unity Lewis number for all species so that the thermal diffusivity is always equal to the mass diffusivity. The algorithm used by the code for pressure–velocity coupling is PIMPLE, a combination of PISO and SIMPLE, using three inner and outer corrections. Firefoam was chosen as it can model shapes more accurately compared to FDS and therefore potentially allow for more accurate convective heat flux prediction for objects with more complex geometry.

In this work, a maximum Courant number of 0.8 was used to solve the time term to ensure numerical stability, following a second-order backward scheme. The convective terms are discretised using the LUST scheme, a scheme with a blending factor of 0.75 central differenced and 0.25 linear upwind scheme. The diffusive terms are discretised using the central differencing scheme with an explicit non-orthogonal correction. As for species mass transport, the terms are discretised using a second-order Total Variation Diminishing (TVD) scheme with Sweby limiter to ensure a bounded solution.

FireFOAM provides several options for the turbulence submodel, combustion model, and thermal radiation modelling. In the section below, the models used in these scenarios are explained.

### Wall Heat Transfer

Coarse grid modelling for convective heat transfer has been developed in FireFoam for industrial-scale study[48, 49]. However, these models have a drawback where it has a specific grid size requirement and straying away from this value requires recalibration of multiple model parameters. In the current work, a wall-resolved simulation is performed where no wall model is used to estimate the convective wall heat transfer. The convective heat transfer is calculated as shown below.

1 $${q}_{c}^{{\prime}{\prime}}={k}_{f}\frac{d{T}_{f}}{dx}$$

where ${q}_{c}^{{\prime}{\prime}}$ is the convective heat flux, k_f_ is the fluid thermal conductivity and, $\frac{d{T}_{f}}{dx}$ is the thermal gradient of fluid and the wall surface temperature in the first cell next to the wall.

It is important to note that for simulations involving laminar flow, the convective heat flux prediction is expected to be poor. This is due to the thinner laminar boundary layer that requires a finer grid size to accurately predict the thermal gradient near the wall.

### Radiation Heat Transfer

For thermal radiation, the Finite Volume Discrete Ordinates Model (fvDOM) is used in the present work to solve the Radiative Transfer Equation in FireFOAM. The method solves the radiation problem by treating the radiation intensity as a function of spatial location and angular direction. The radiation is assumed to be a non-scattering, non-absorbing, and optically thin medium in the current study. The radiation emission is modelled via the radiative fraction approach to avoid the uncertainty involved in modelling the turbulent radiation interaction (TRI), soot modelling and reducing the dependency of grid size to model radiation accurately due to filtered temperature, ${\widetilde{T}}^{4}$. No soot or spectrum modelling was employed as the current study intend to focus on flow and heat transfer by convection.. The radiative fraction approach assumes that a portion of the HRR of fuel combustion is converted into thermal radiation, as shown below

2 $$\nabla \cdot {q}_{R}^{{\prime}{\prime}}=\chi {q}_{comb}^{{\prime}{\prime}{\prime}}$$

where ${q}_{R}^{{\prime}{\prime}}$ is the radiation heat flux, $\chi$ is the radiative fraction constant and ${q}_{comb}^{{\prime}{\prime}{\prime}}$ is the volumetric HRR due to combustion. The radiative fraction, *χ*, used is 0.27, which is within the range found experimentally [50]. Sensitivity analysis performed using a radiative fraction of 0.27 and 0.35 was found to have a negligible difference in the current study. Finally, the number of solid angles used for the simulations is 112. Simulation results show that using a lower solid angle may result in an inaccurate prediction of wall heat flux due to the “ray effect”.

In this study, the total incident heat flux on the wall is evaluated with the following expression:

3 $${q}_{t}^{{\prime}{\prime}}={q}_{c}^{{\prime}{\prime}}+{\alpha q}_{in}^{{\prime}{\prime}}$$

where ${q}_{t}^{{\prime}{\prime}}$ is the total incident heat flux $\alpha$ is the absorptivity coefficient of the material and ${q}_{in}^{{\prime}{\prime}}$ is the incident radiation heat flux.

### Turbulence

There are several turbulence models available in FireFoam, such as the Smagorinsky model, *k*-equation eddy viscosity model (k-equation model), and wall adapting local eddy-viscosity (WALE) model.

In the present study, the SGS turbulence was modelled with wall adapting local eddy-viscosity (WALE) model, as it predicts the SGS kinetic energy, ${k}_{sgs}$, and SGS eddy viscosity,${\nu }_{sgs}$, better in the near-wall region compared to the default k-equation model used in FireFOAM [49, 51]. The ${\nu }_{sgs}$ is modelled using the model constant, C_w,_ = 0.55 based on [52]. The SGS turbulent kinetic energy, ${k}_{sgs}$ and the rate of dissipation of SGS turbulent kinetic energy, ${\varepsilon }_{sgs}$, in WALE calculated using the model constants, C_k_ = 0.29 and C_E_ = 1.048 based on [52].

However, to ensure the WALE model implemented is suitable for cavity flow, both Smagorinsky and k-equation eddy viscosity models are used in Scenario 1 Flow and Scenario 2 Heat to compare the differences between the models.

In the Smagorinsky model, the *ν*_*sgs*_ is modelled using the model constant C_s_ = 0.1 based on [53]. To obtain *k*_*sgs*_ and ${\varepsilon }_{sgs}$, the Smagorinsky model constant of C_k_ and C_E_ have a coefficient of 0.05 and 1.048, respectively.

For the k-equation model, the ${\nu }_{sgs}$ is calculated using the model constant of 0.05. To obtain ${\varepsilon }_{sgs}$ the model constant of C_E_ = 1.048 is used.

Both Smagorinsky and k-equation eddy viscosity models have been shown to overpredict near-wall ν_sgs_ and k_sgs_. To mitigate this issue, the Van Driest wall damping function was used to suppress ν_sgs_ near the wall by altering the LES filter size. Refer to the online appendix for more details on the turbulence submodels.

### Combustion

The modified eddy dissipation combustion model (EDM) proposed by Ren et al. is the most commonly used combustion model for FireFoam and was used in the present study[52]. The modified EDM expressed the fuel mass reaction rate as:4where *ρ* is the gas density, ${{k_{sgs} } \mathord{\left/ {\vphantom {{k_{sgs} } {C_{EDC} \varepsilon_{sgs} }}} \right. \kern-0pt} {C_{EDC} \varepsilon_{sgs} }}$ is the turbulent mixing timescale with C_EDC_ the model coefficient C_EDC_ = 4, ${{\Delta^{2} } \mathord{\left/ {\vphantom {{\Delta^{2} } {C_{Diff} \alpha }}} \right. \kern-0pt} {C_{Diff} \alpha }}$ is the molecular diffusion timescale with C_Diff_ the model coefficient C_Diff_ = 4, $\alpha$ is the thermal diffusivity, $\tilde{Y}_{F}$ is the fuel mass fraction, $\tilde{Y}_{{O_{2} }}$ is the oxygen mass fraction, and *r* is the stoichiometric oxygen-to-fuel mass ratio. The combustion model coefficients are based on work done by Ren et al., where vertical wall fire is simulated [10].

The model assumes one-step complete combustion, infinitely fast, and considers irreversible chemical reactions. It assumes that in the turbulent region, the fuel–air mixing is controlled by turbulent mixing, while in the laminar region (i.e., viscous sublayer of the wall), it is controlled by molecular diffusion.

### Computational Domain

For all Scenarios, the cells are hexahedra structured mesh generated using the OpenFOAM default utility. All cells are generated as cubic cells except those near the wall where the cell direction normal to the wall, Δy, is halved. In Scenario 1 Flow, the domain is based on the experiment by Hussain et al. [35]. The domain is 200 × 100 ×  63.5 mm^3^. The boundary condition of the simulation for both streamwise and spanwise was set as periodic boundaries. Note that while periodic boundaries are not suitable when a fire is introduced as the fire structure would not be modelled accurately near the boundary, the purpose of using periodic boundaries in this scenario is to validate the model capabilities for fluid flow physics without increasing the required computational resources unnecessarily. For both the top and bottom of the domain, a no-slip boundary was applied. The velocity in the domain is perturbed to generate the initial turbulent field, and the mean velocity in the domain was set to 5.77 m/s. The perturbation of the turbulent field is performed using the boxTurb tool available in OpenFOAM where divergence-free turbulence conforming to a given turbulence energy spectrum is created. Grid sensitivity was performed using three different grid sizes Δy = 0.78, 0.62, and 0.50 mm. The analysis found that the difference between Δy = 0.62 mm and 0.50 mm is negligible, with Δy = 0.78 mm slightly underpredicting the velocity fluctuation. Hence, a grid size of Δy = 0.62 mm was chosen in this Scenario.

For Scenario 2 Heat, a similar numerical domain for Scenario 1 Flow was used with the cavity width changed to replicate the experiment [33]. The top and bottom were set as no-slip with a fixed temperature of 46°C and 30°C, respectively. The velocity in the domain is perturbed, with the bulk velocity set to 4.66 m/s. Grid sensitivity was analysed using the same grid sizes of S1 Flow. The setup was found to be grid insensitive after grid size Δy = 0.62 mm.

As for Scenario 3 Buoyancy, the domain is based on the experiment by Miyamoto et al. [37]. The numerical domain is 600 × 6117 × 960 mm^3^. Due to the symmetrical setup of the experiment, only one cavity was simulated to reduce computational time. The domain behind the adiabatic wall was also removed to reduce the computational resources required, as analysis found the removal had a negligible effect on the result. The open boundary and wall boundary of the domain are shown in Online Appendix 3. A total of six different cases were investigated with cavity widths of W = 50 mm, 100 mm, and 200 mm for each wall heat flux of 104 W m^−2^ and 208 W m^−2^. The convective heat flux at the heated wall was prescribed as 83.2 W m^−2^ and 166.4 W m^−2^, respectively, as estimated in the experiment [37]. The boundary condition of the heated wall is as follows:

5 $$-{k}_{f}\frac{d{T}_{f}}{dx}=q+{q}_{r}$$

where q is the prescribed heat flux set in the experiment by Miyamoto et al. [37] and q_r_ is the radiation heat flux.

For setup where the wall heat flux is 104 W m^−2^, the heated wall temperature along its height was measured, while for configuration with 208 W m^−2^, the velocity along the cavity was measured. The grid sensitivity analysis was performed with grid sizes of Δy = 4 mm, 2 mm, and 1 mm, and the result showed that the solution becomes grid insensitive at Δy = 2 mm.

**Figure 4:**
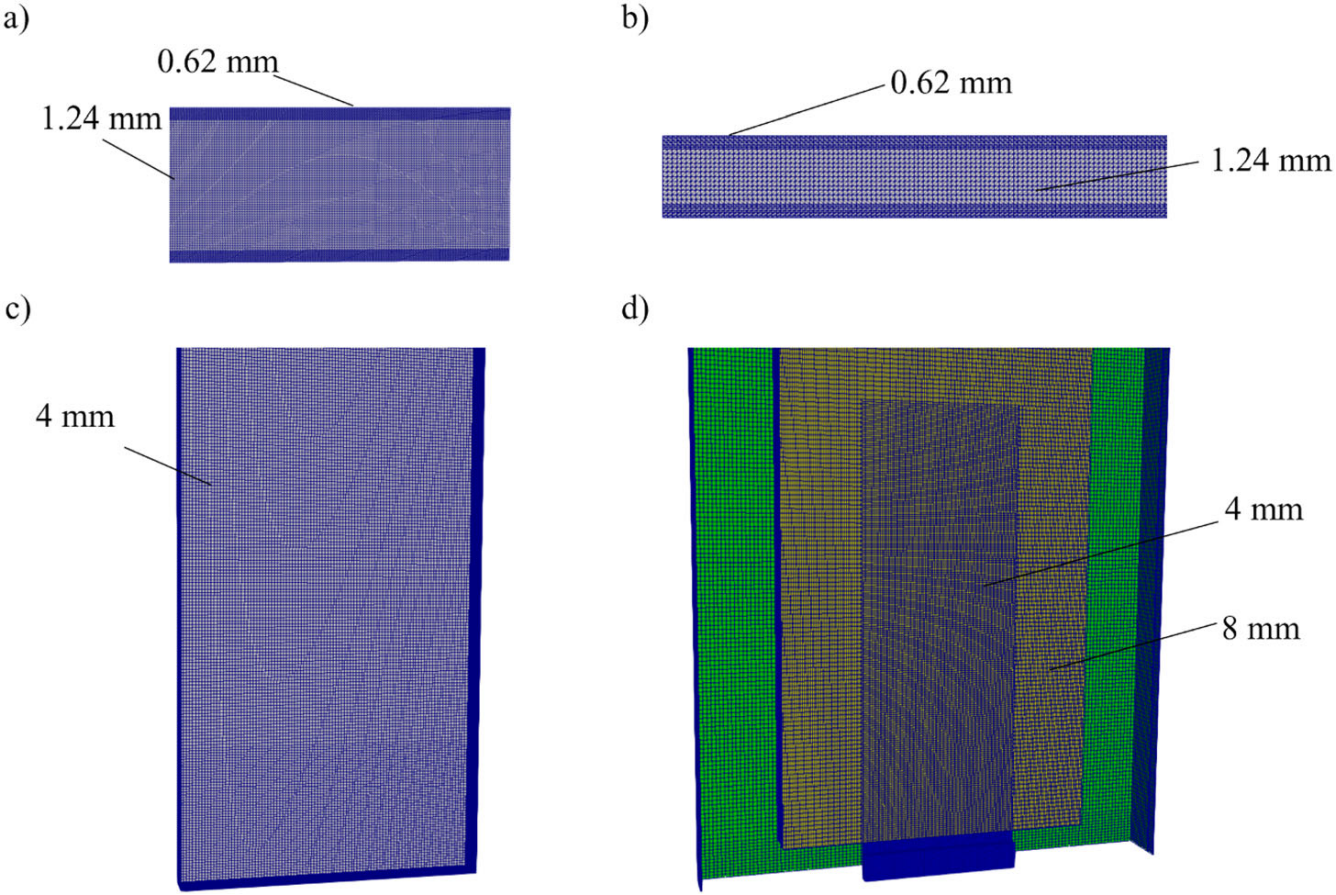
Final Numerical configuration of Scenario 1, 2, 3, and 4, (a, b, c and d). Grid size in the respective refinement zone is as shown in the Figure

The numerical domain of Scenario 4 Combustion, as shown in Figure 4, is 1200 × Y × 2100 mm^3^, where Y is 240, 250, 260, and 300 for cavity widths W = 40 mm, 50 mm, 60 mm, and 100 mm respectively. The thermal properties of the wall are defined as are taken as defined by Livkiss [23]. The boundary conditions at the wall are expressed as follows:

6 $$k_{s} \frac{{dT_{s} }}{dx} = k_{f} \frac{{dT_{f} }}{dx} + \alpha q^{\prime\prime}_{in} - \varepsilon q^{\prime\prime}_{em}$$

where k_s_ is the thermal conductivity of the solid, ${{dT_{s} } \mathord{\left/ {\vphantom {{dT_{s} } {dx}}} \right. \kern-0pt} {dx}}$ is the solid thermal gradient, α and ε are the absorptivity and emissivity coefficients, both taken as 1 due to the reported soot on the wall [23], $q^{\prime\prime}_{in}$ is the incident radiation heat flux, and $q^{\prime\prime}_{em}$ is the emitted radiation heat flux. Grid refinement is performed near the around the burner to obtain more accurate prediction of the flame structure. The refinement of the grids is half the size of the non-refined area. Grid sensitivity analysis was performed on grid sizes of Δy = 4 mm, 2 mm, and 1 mm. The results show that in turbulent regions, the wall heat flux is grid-insensitive even at Δy = 4 mm. However, at laminar regions, the wall heat flux solution is highly sensitive to grid size, with 70% and 40% differences compared to Δy = 1 mm for Δy = 4 mm and Δy = 2 mm, respectively. As the majority of the analysis was done in turbulent regions, the grid size of Δy = 2 mm was chosen in this study to reduce computational time.

The total numerical cells used in each case are shown in the table below (Table 1):

**Table 1:** Total Number of Grid Cells Within Each Scenario

Scenarios	Δy, mm	Total numerical cells
S1 Flow	0.62	1,400,000
S2 Heat	0.62	480,000
S3 Buoyancy	2.00	3,898,000
S4 combustion	2.00	702,640–1,073,242

Note that numerical cells for Scenario 4 Combustion differ depending on the cavity width

## Numerical Results

### Scenario 1 Flow

**Figure 5:**
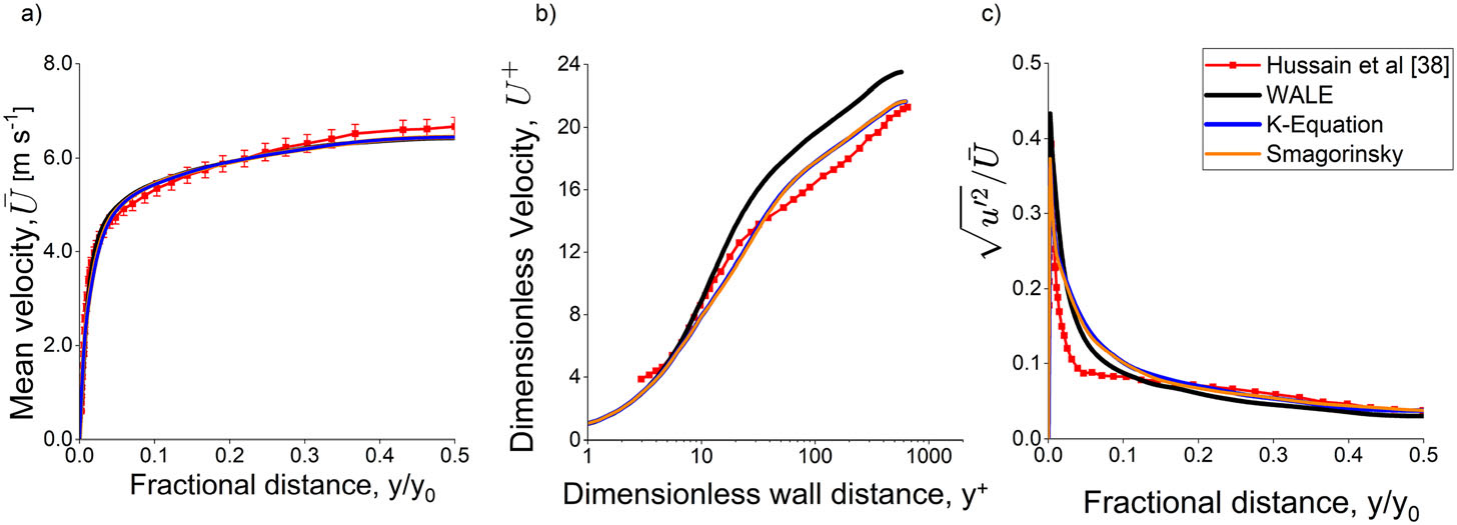
Predicted Scenario 1 Flow (a) wall-normal variation of the mean velocity profile (b) log law velocity profile, where y^+^ is the dimensionless wall distance (c) Dimensionless velocity intensity profile. The simulation result shows that all turbulence submodels predict flow velocity in a narrow cavity well

The analysis shows that predictions of the mean velocity profile, friction velocity, and turbulent velocity fluctuation of the three turbulence models fit the experimental data with reasonable accuracy, as presented in Figure 5. The predictions are within 10% of the experimental measurements for all submodels.

All submodels predict the fluid flow to a good degree of accuracy, although the k-equation and Smagorinsky submodels perform slightly better than WALE at predicting the log law velocity profile, with average errors of 7.6%, 7.9%, and 12%, respectively. This is attributed to the Van Driest damping function that helps predict velocity gradient correctly and, therefore, better friction velocity prediction [54].

It should be noted that for the U^+^ predicted for y^+^ < 4 is not reported by Hussain et al. [35]. Therefore, simulation result for y^+^  < 4 was not compared to experimental result.

The velocity fluctuations of different turbulence submodels are compared to the experimental measurement in Figure 5c. The predicted velocity fluctuations by the three models at the centre of the channel and the peak value were similar to the experimental result. However, all three models overpredict at the near-wall region by an average of 30%, with both k-equation and Smagorinsky submodels predicting the fluctuations better than the WALE model near the plates before overpredicting the fluctuations closer to the centre of the channel. As aforementioned, using the Van Driest damping function for both k-equation and Smagorinsky models may have improved the prediction of this channel flow.

### Scenario 2 Heat

**Figure 6:**
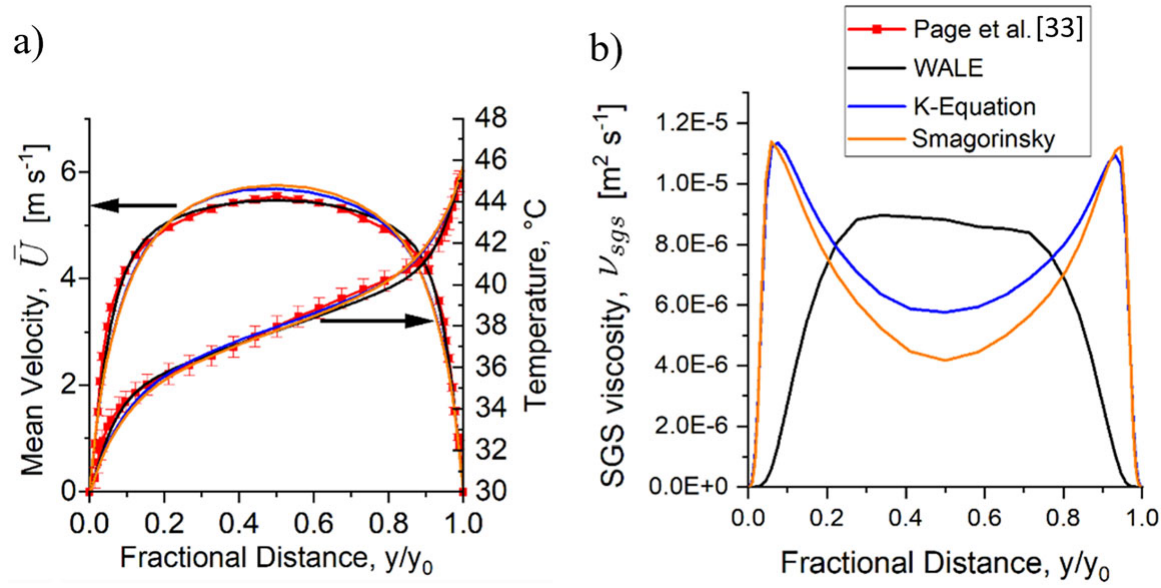
Predicted results of Scenario 2 Heat where (a) The mean velocity and temperature profiles were predicted well by all turbulence models. (b) The vanishing value of SGS viscosity at the wall was only predicted well by WALE

For Scenario 2 Heat, all three turbulence submodels predict the velocity and temperature profiles with a maximum error of about 20%, as shown in Figure 6a. The WALE submodel predicts the heat flux slightly better than k-equation and Smagorinsky. The wall heat flux measured in the experiment is 150 W m^−2^. The WALE predicted a heat flux of 102 W m^−2^ while k-equation and Smagorinsky both predicted 93 W m^−2^. The reason for this discrepancy is due to both k-equation and Smagorinsky submodels tend to take an incorrect value of subgrid-scale viscosity near the walls, where it increases substantially before a sudden reduction to zero due to Van Driest damping function, as shown in Figure 6b. This meant that k-equation and Smagorinsky submodels predicted a more turbulent flow near the wall. This phenomenon was observed by Yuen et al. [55] where they found turbulent generation near a wall in a compartment fire is greater for models that require damping function. In contrast, the WALE model could predict the vanishing value of ν_sgs_ near the wall without a damping function, and hence a more accurate flow at the wall.

The simulation results were non-dimensionalised and compared to the DNS results by Lyon et al. [56] to validate the accuracy of the fluctuating temperature predictions. DNS simulation result was l due to the lack of reliable temperature measurement near the wall at high flow speed [57].

Two different non-dimensionalised numbers were compared, the mean temperature distribution ${\overline{T} }^{+}/Pr$ and the r.m.s temperature distribution ${{\theta }{\prime}}^{+}/Pr$, shown in (7)and (8), respectively

7 $$\frac{{\overline{T} }^{+}}{Pr}=\frac{{T}_{w}-\overline{T}}{{T }_{\tau }}\times \frac{1}{Pr}$$

8 $$\frac{{{\theta }^{\prime}}^{+}}{Pr}=\frac{{\theta }^{\prime}}{{T}_{\tau }}\times \frac{1}{Pr}$$

where T_w_ is the wall temperature, $\overline{T }$ is the mean temperature, and ${T}_{\tau }$ is the friction temperature defined as wall heat flux divided by friction velocity, ${q}_{w}/{U}_{\tau }$, ${\theta }^{\prime}$ is the root mean square temperature fluctuation and Pr is the Prandtl number.

**Figure 7:**
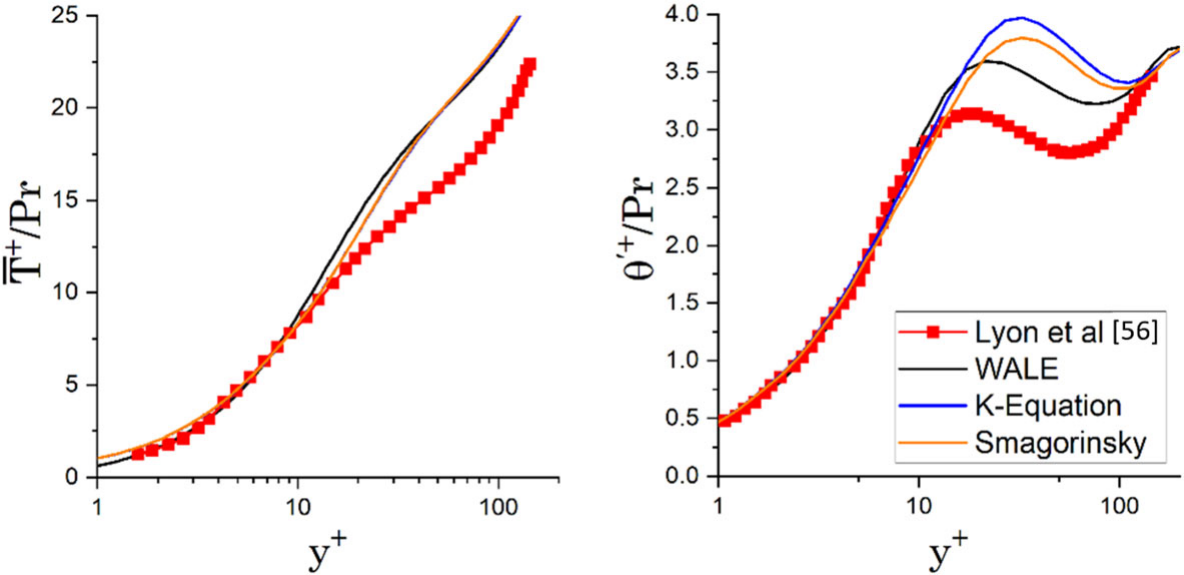
Prediction of Scenario 2 Heat of (a) non-dimensional mean temperature distribution. (b) non-dimensional r.m.s temperature distribution from the wall to the centre of the cavity

Figure 7 shows that all three turbulence submodels predict well the mean temperature with an error lower than 20% at the centre, but when comparing the temperature fluctuation WALE gave a closer prediction with an average error of 6.7%.

The simulation results in Scenario 2 Heat show that WALE predicts better than Smagorinsky and K-equation due to ν_sgs_ being predicted better near the wall, where it is vital in the prediction of near-wall combustion and wall heat flux [49, 55]. WALE was chosen for Scenario 3 Buoyancy and Scenario 4 Combustion as it potentially improves near-wall predictions as it predicts the vanishing ν_sgs_ near the wall without needing a damping function that does not consider the local effect in a complex flow.

### Scenario 3 Buoyancy

**Figure 8:**
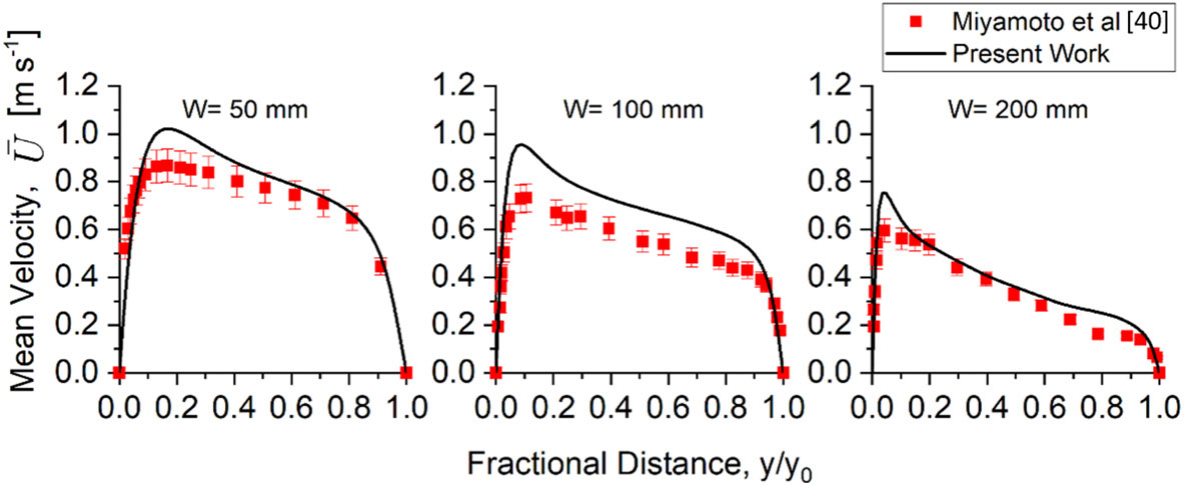
Comparison of Scenario 3 Buoyancy experimental and predicted velocity profiles near wall for cavity widths of W = 50 mm, 100 mm, and 200 mm at the height of 3865 mm for q_w_ = 208 W. The model predicts the general trend, although velocity tends to be overestimated near wall for all cavity widths

When the wall heat flux is 208 W m^−2^, the results show that the model predicts the velocity profile due to buoyancy within an average error of 30% for all cavity widths, as shown in Figure 8. The uncertainty of the convection heat flux generated from the heated wall in the experiment as reported by Miyamoto et al. [37] may partially explain the differences, although it is unlikely that it is the main reason for the discrepancies.

**Figure 9:**
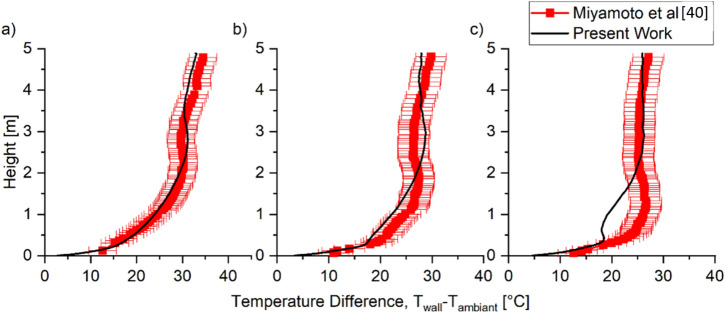
Comparison of Scenario 3 Buoyancy experimental and predicted mean temperature of the heated wall at cavity widths of (a) 50 mm, (b) 100 mm, and (c) 200 mm cavity for q_w_ = 104 W. The model captured the general trend, but local maxima tend to be predicted slightly higher up

When the heated wall generates a heat flux of 104 W m^−2^, the local maxima of the heated wall temperature were predicted higher than the experiment measurement by 20%, although the general temperature trend was captured, as shown in Figure 9. This overprediction of the local maxima could be due to the model’s ability to predict convective heat transfer at the laminar region. As the boundary layer thickness is smaller for laminar flow than turbulent flow, a finer grid size is needed to give better convective heat transfer at the laminar region. The simulation results showed that the convective heat transfer coefficient on the wall was in the range of 3 to 20 W m^−2^ K^−1^.

**Figure 10:**
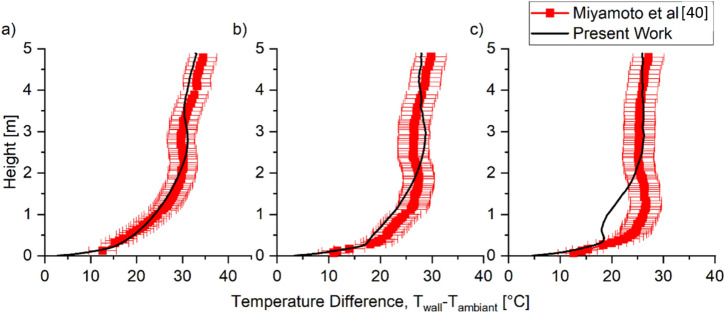
Comparison of Scenario 3 Buoyancy experimental and predicted turbulent characteristics of velocity fluctuation (a) and (b) and non-dimensional temperature fluctuation profile (c) when q_w_ = 104 W at the height of 3865 mm. The model was able to predict the general trend of the turbulent characteristic

The turbulent characteristic for the case of heat flux of 104 W m^−2^ was also compared to the experimental result for cavity widths of 100 mm and 200 mm. The simulations predict both velocity and temperature fluctuation profiles and temperatures to a reasonable degree with errors below 35%, as shown in Figure 10. However, similar to the prediction of the velocity profile, the model tends to overpredict the velocity fluctuation near the heated wall for the cavity width of 100 mm. At the centre of the cavity between the two plates, the vertical velocity fluctuation, u’, was underpredicted for both cavity widths. As for the normal velocity fluctuation v^’^, the model overpredicts for 100 mm cavity width with an average error of 40%, while for 200 mm cavity width, the model predicts with an average error of 25%. One potential reason for these errors is the need for a finer grid size to predict the convective heat transfer at the laminar region more accurately. The temperature fluctuation prediction by the model was found to be accurately predicted throughout the cavity.

These results indicate that the model predicts the general trend of both wall temperature and velocity profile in a cavity due to natural convections with an average error of 40%. The results show that the introduction of buoyancy significantly increased the complexity of the problem with respect to Scenario 2 and the difficulty of predicting fluid dynamics in the cavity. Therefore, it is essential to be aware that while the model can accurately predict the general trend the errors are not negligible.

### Scenario 4 Combustion

#### Flame Height

**Figure 11:**
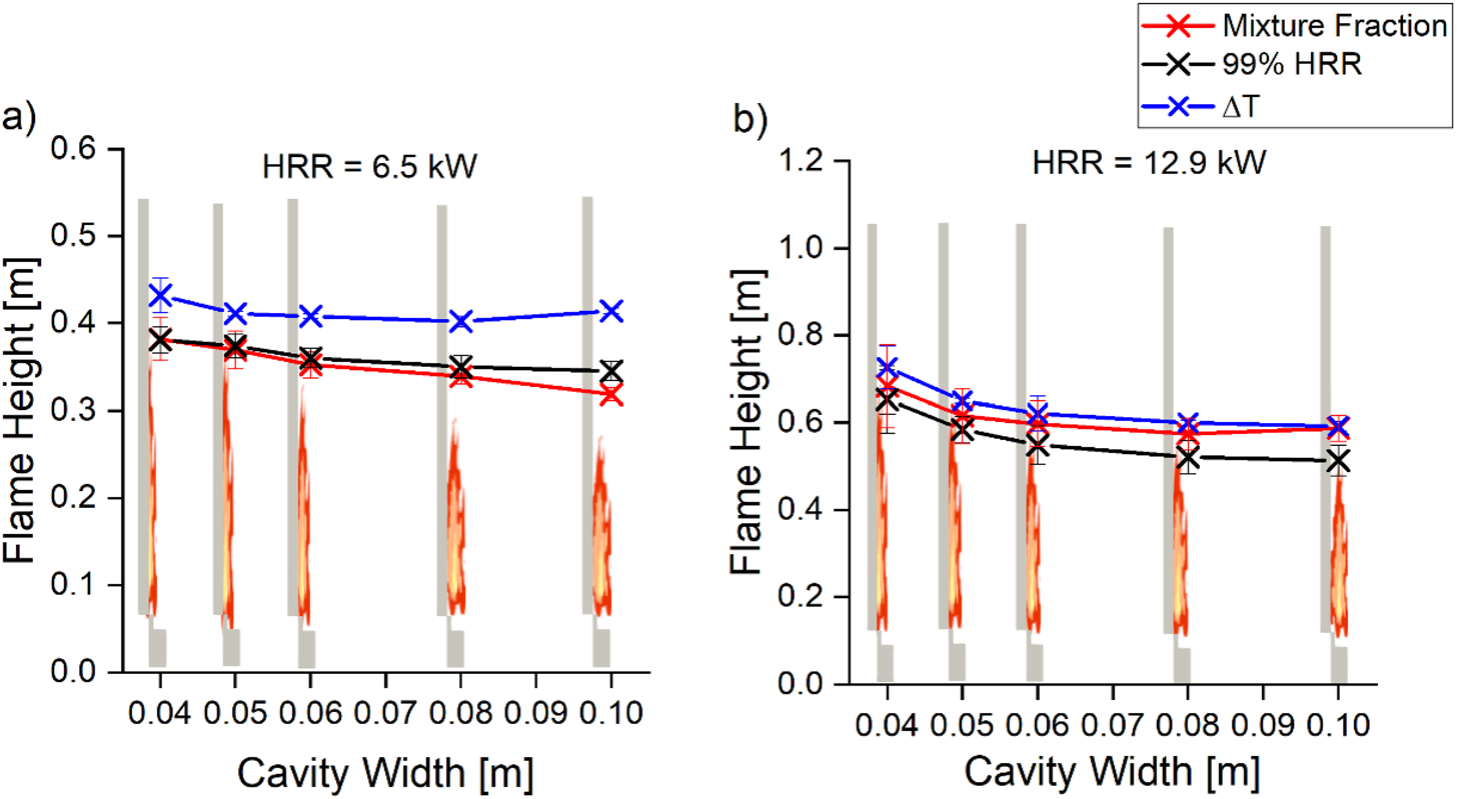
Comparison of Scenario 4 Combustion flame height using three different criteria: stoichiometric Mixture Fraction, 99% cumulative HRR and ΔT = 550 K. The comparison shows mixture fraction and HRR agree well

**Figure 12:**
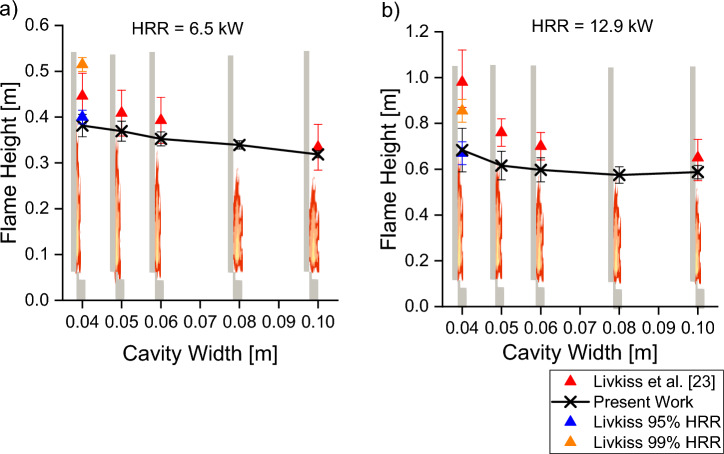
Comparison of Livkiss’s experimental and Scenario 4 Combustion flame height prediction where burner HRR is (a) 6.5 kW and (b) 12.92 kW. The model consistently underpredicts the flame height, but the reduction of flame height with increasing cavity width was predicted

The flame heights predicted by our simulations are compared to the experimental and simulation results by Livkiss et al. [23, 30]. Flame height was defined using visual analysis and cumulative HRR, respectively. In our work, flame height was first compared with three criteria used in past literature [58]: stoichiometric mixture fraction, 99% cumulative HRR and temperature difference of 550°C with the surrounding air temperature, ΔT. The median flame height between 30 s and 40 s was compared and shown in Figure 11. The comparison shows that the difference in flame height measured using stoichiometric mixture fraction and 99% cumulative HRR criteria is minimal, while flame height predicted using ΔT criteria is higher by up to 25%. The stoichiometric mixture fraction criterion was chosen to measure the flame height in this work for the sake of simplicity as it is a function present within the FireFOAM library. The flame heights are then compared to Livkiss’s, as shown in Figure 12.

Figure 12 shows that for the two burner HRR, the model tends to underpredict the flame height. Analysis shows that flame heights predicted by the model were more accurate for lower burner HRR, where underpredictions are below 20% of the experimental flame height. In comparison, at higher burner HRR, the flame heights were underpredicted by 40%. The present predictions are within 20% of the prediction by Livkiss, where flame heights of cavity width of 40 mm were simulated and underpredicted. One potential reason for the underprediction was the infinitely fast combustion model that might result in an underprediction of flame height in a well-ventilated condition. This is due to fuel reacting with oxygen immediately at contact, which resulted in the majority of the combustions occurring in the first grid cell next to the burner [59].

**Figure 13:**
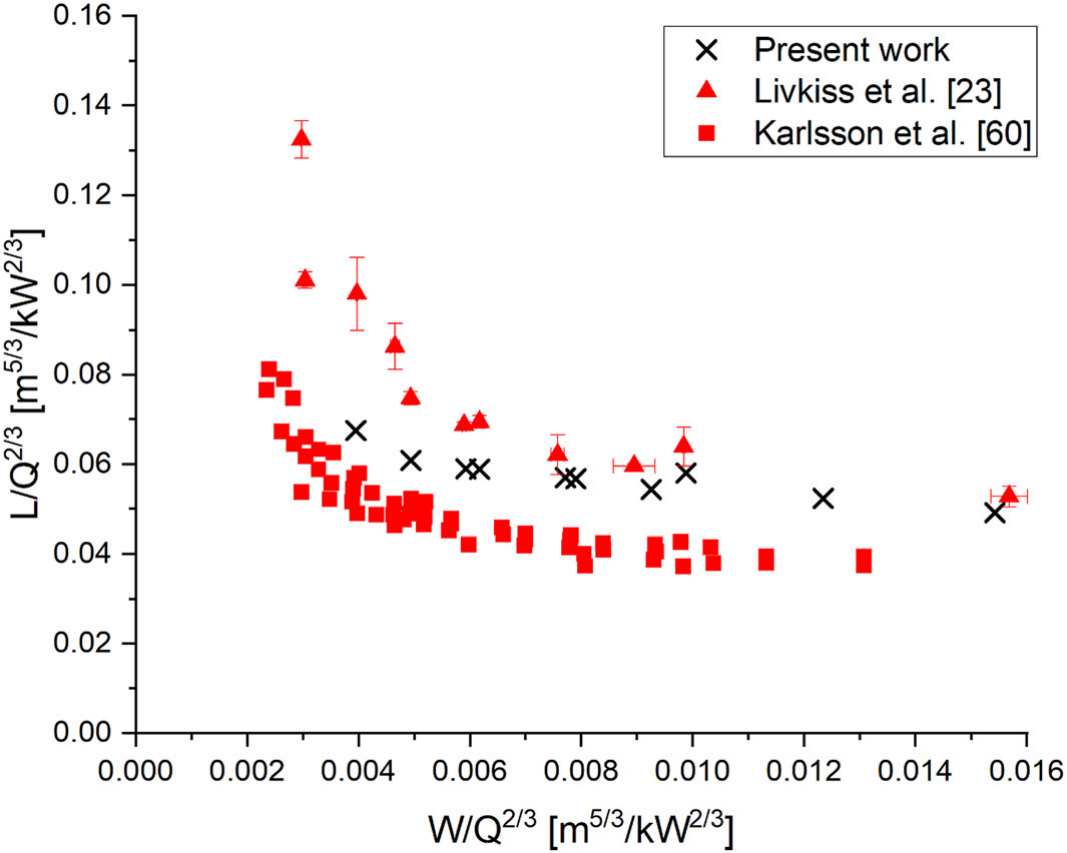
Comparison of Scenario 4 Combustion flame height with both Livkiss et al. and Karlsson et al. experimental measurements [23, 60]. The model prediction is within the measurement of both data sets, suggesting that the suitable for predicting flame height for different cavity configurations

However, while flame heights were underpredicted in both HRR, the model captured the general trend of flame height reducing with an increasing cavity width. The flame height predictions were also compared with other narrow cavity fires by comparing the flame height per HRR and cavity width per HRR. Figure 13 shows the predicted flame height when compared to both Livkiss et al. and Karlsson et al. experimental measurements [23, 60]. The results show that the model could potentially be extrapolated to study different facade configurations.

#### Exit Velocity

**Figure 14:**
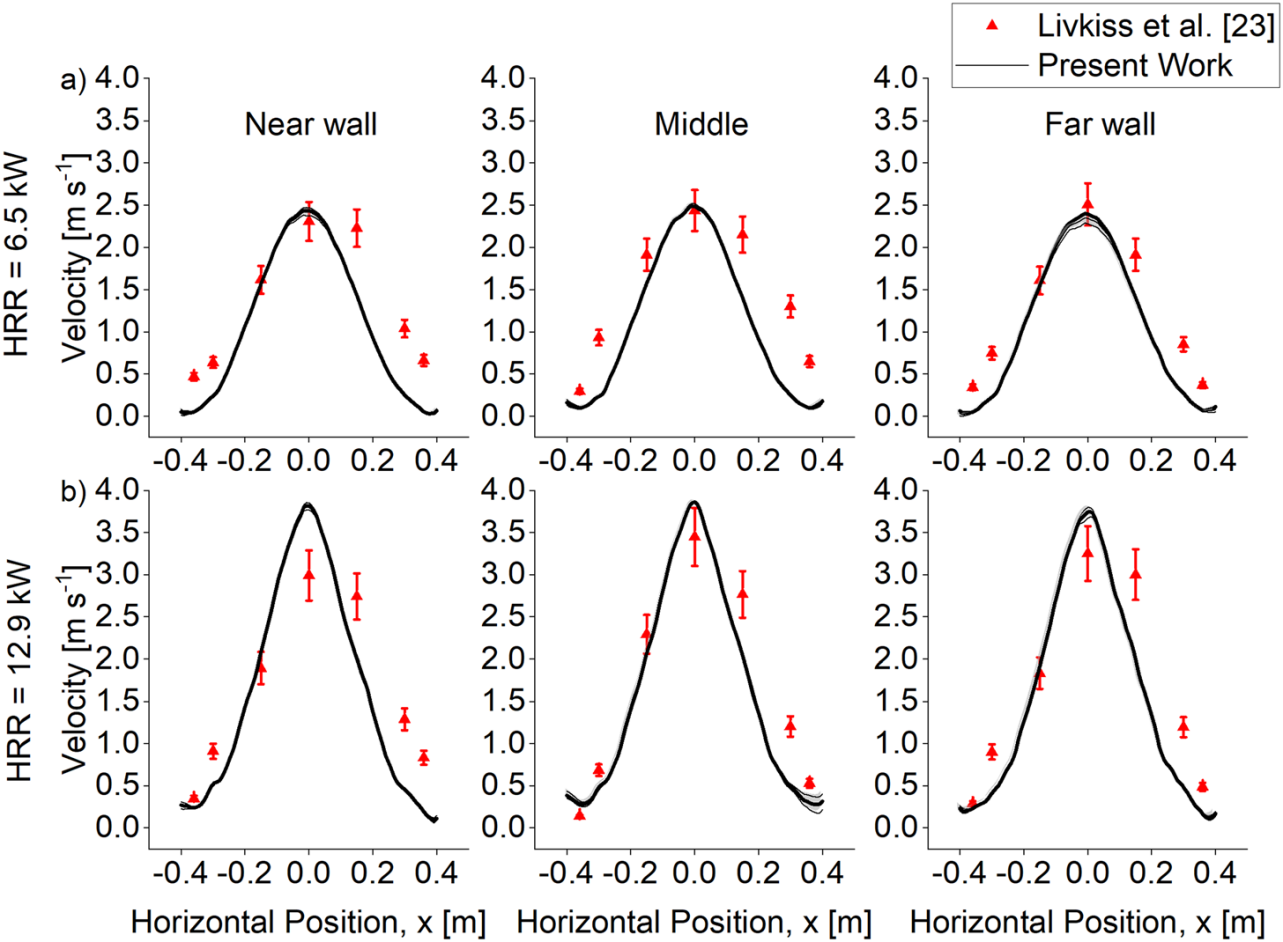
Comparison of S4 Combustion result and experimental upward exit velocity data at various locations at the cavity for a cavity width of 40 mm with Burner HRR of (a) 6.5 kW and (b) 12.92 kW. The simulation result captures the general trend of the experimental data, albeit slightly underpredicted

The upward exit velocity is measured The predicted upward exit velocities for both burner HRR are 1800 mm above the burner and were averaged over 10 s. The velocities were found to be underpredicted at all locations, but the general trend is correct, as shown in Figure 14. Note that the experimental results are not symmetrical because the placement of the gas burner may be unsymmetrical, as reported by Livkiss et al. [23]. The predicted maximum velocity was found to have an error within 4% for HRR 6.5 kW, while for HRR 12.9 kW, the error was found to be within 20% However, the velocity prediction at the side was found to be underpredicted with an average error of 51%. Given the uncertainty in the measurements and the general trends captured by the model, the model’s capability to predict peak exit velocity in a facade cavity fire was satisfactory.

#### Total Incident Heatflux to the Near Wall

**Figure 15:**
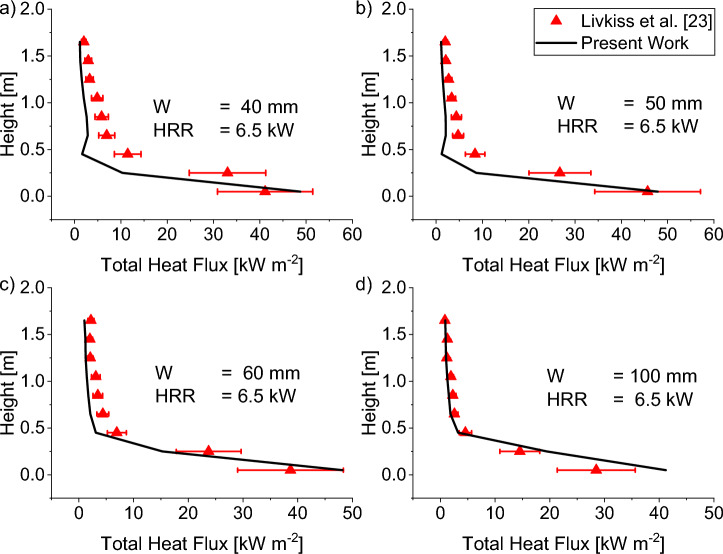
Comparison of experimental total incident heat flux to Scenario 4 Combustion prediction of HRR = 6.5 kW for a cavity width of (a) 40 mm, (b) 50 mm, (c) 60 mm, and (d) 100 mm. A good match to experimental data was found qualitatively and quantitatively for varying cavity widths

**Figure 16:**
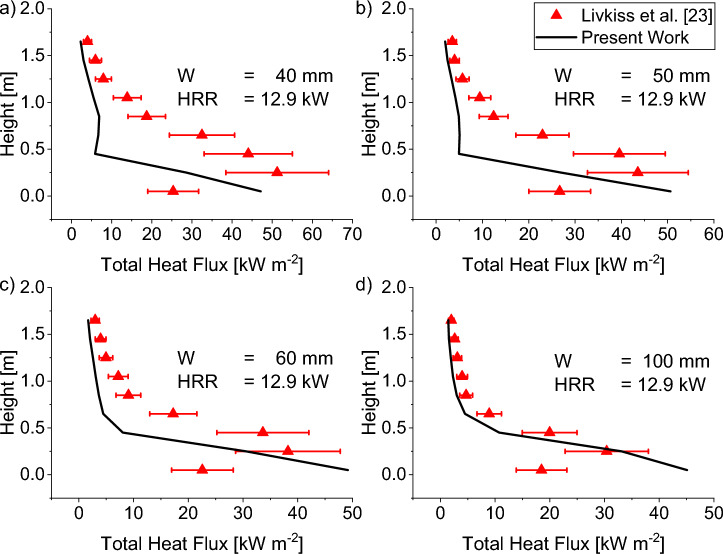
Comparison of experimental total incident heat flux to Scenario 4 Combustion prediction of HRR = 12.9 kW for a cavity width of (a) 40 mm, (b) 50 mm, (c) 60 mm, and (d) 100 mm. The prediction was found to match the experimental data less at lower elevations

The comparison of total incident heat flux, calculated using (3), along the centreline of the near wall for both 6.5 kW and 12.92 kW HRR with varying cavity widths is shown in Figures 15 and 16. The heat flux presented in these figures is defined similarly to Livkiss [30], where it is the sum of convective heat transfer and incident radiation heat flux. The simulation results show a better prediction for HRR 6.5 kW HRR with an average error of 37%. In contrast, for HRR 12.9 kW, the predicted heat flux is less accurate, with an average error of 45% and a maximum error of 90%.

**Figure 17:**
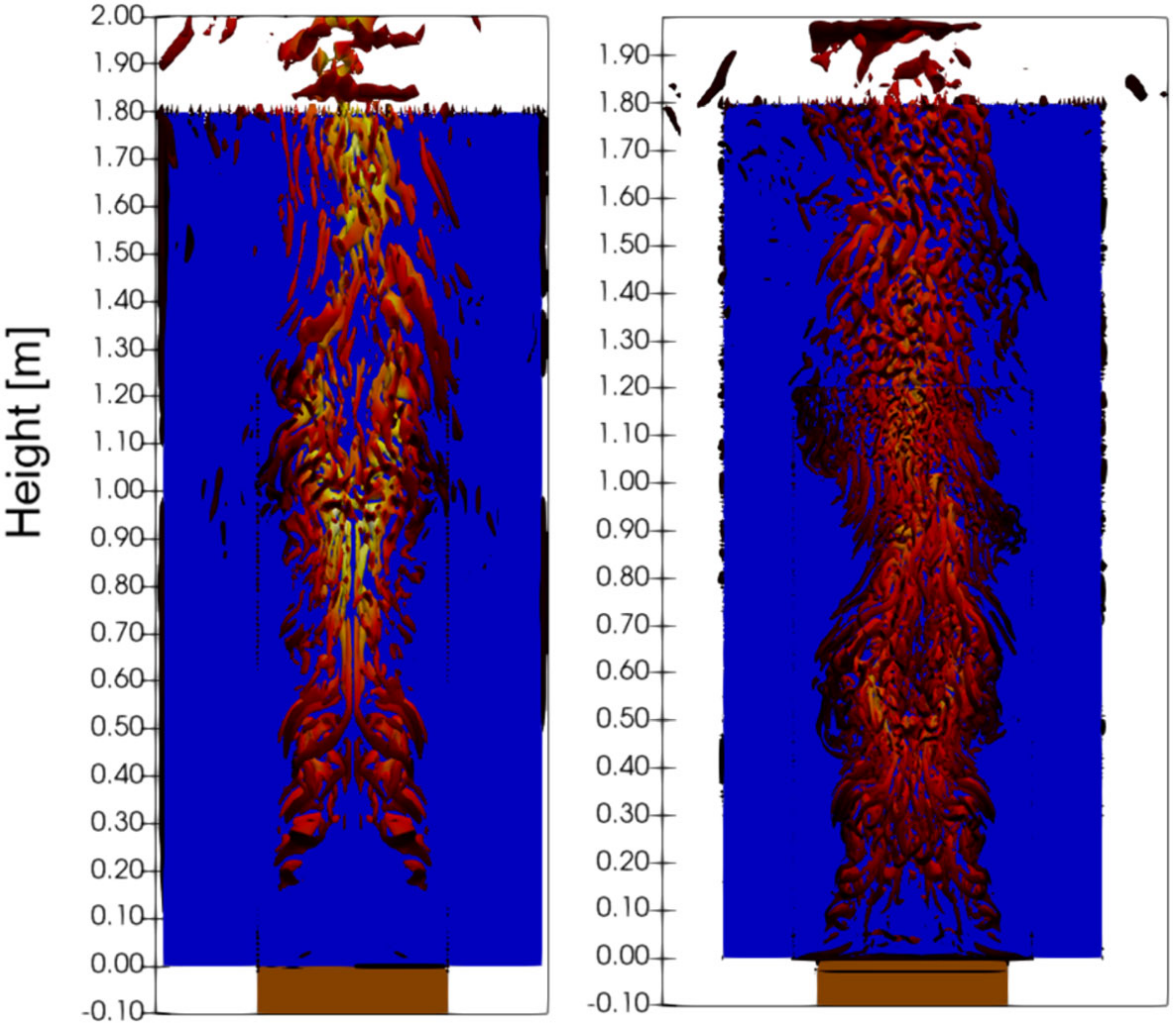
Vortical structure of the flame using Iso-contour of Q, Q = 1000 s^−1^- based on Ren et al.’s [49]. (Left) The iso-contour of Q using the Medium mesh shows turbulence inception occurs around 0.45 m. (Right) The iso-contour of Q using the Fine mesh shows turbulence inception occurs around 0.20 m

Plausible explanations for these errors are flame lift-off that cannot be simulated with the EDM combustion model. The lack of lift-off may explain the over-predictions of heat flux at lower elevations and underprediction at higher elevations. The underprediction of flame height also results in lower radiative heat flux at a higher elevation. The error could also be attributed to the model’s lack of ability to predict laminar-turbulence transition and convective heat flux in the laminar accurately. Improvement to the accuracy of laminar-turbulence transition and convective heat flux could be made using a fine grid, as shown in Figure 17. The simulation prediction found that for a cavity width of 0.4 m, the turbulence transition occurs approximately around z = 0.5 m for a grid size of Δy = 2 mm and transition around z = 0.2 m when a finer grid size of Δy = 1 mm is used. This resulted in an increased convective heat flux prediction of the convective heat flux for 0.2 m < z < 0.5 m for the grid size of Δy = 1 mm.

**Figure 18:**
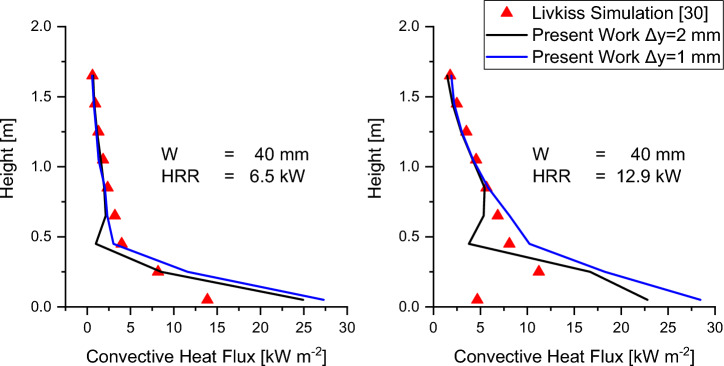
Comparison of Convective Heat Flux predicted by different grid sizes in Scenario 4 Combustion and Simulation by Livkiss. Results show that a fine grid is needed for FireFoam to predict the laminar convective heat transfer more accurately

Figure 18 shows the comparison of the present work with Livkiss’s FDS model [29], which calculates convective heat flux using the default FDS wall function [61]. Livkiss’s result shows that the predicted convective heat flux is similar in the turbulent region but differs in the laminar region. The inaccuracy in the predicted convective heat flux at the laminar region near the wall was also previously observed by Ren et al. [49].

An accurate prediction of the laminar-turbulent transition point and laminar convective heat flux could be achieved by having a much finer grid, which is not possible in the current case due to the required computational resources. However, it is important to note laminar region is generally small (typically 10–20 cm) [62] and engineering wall fire problems are typically in the turbulent. This means the current model can still be useful to study cavity fire where the fire is predominantly turbulent.

## Conclusions

We study the ability of the FireFOAM model to predict cavity fire with varying widths. The present work includes fluid flow, heat transfer, buoyancy and combustion physics mechanisms. Since facade cavity fire is a multiphysics process where different physical phenomena interact with each other to produce a complex problem. The present work validates each physical phenomenon separately in a narrow cavity configuration. Current cavity fire models are compared to experimental result with all physical phenomena at once. But the model may be affected by the compensation effect where a good result could be obtained due to multiple poorly predicted physical phenomena. To limit the compensation effect of the model, the current work performs a step-by-step validation to ensure each physical phenomenon is predicted well independently in a narrow cavity configuration.

Simulation results from Scenario 1 Fluid and Scenario 2 Heat show that the presented model can capture both fluid flow and heat transfer physics accurately. Analysis from both scenarios indicates that WALE turbulence model is more appropriate for a cavity fire due to the walls’ proximity. The result shows that WALE predicts the temperature gradient and velocity profile in a turbulent flow more accurately than the k-equation and Smagorinsky models in a narrow cavity. In addition, the ability of WALE to predict the vanishing value of SGS viscosity and kinetic energy without relying on a damping function meant that it could predict convective heat flux and combustion near the walls better.

The model’s ability to predict better buoyancy physic was also compared to experimental result. The simulation results show that the model slightly overpredicts the airflow in the cavity. The results also show that the model was able to predict the general trend of the heated wall temperature with reasonable well, indicating that the model is capable of simulating buoyancy in a narrow cavity.

Simulation results from Scenario 4 Combustion show that while the model underpredicts the flame height for various cavity widths, the model captures the general trend of the decreasing flame height with increasing cavity width. The simulation was also able to capture the exit velocity well. The main challenge in Scenario 4 Combustion was for the model to accurately predict the total heat flux due to fuel combustion. The results show that the total heat flux at the near-wall predicted by the model is better for burner HRR of 6.5 kW, but for 12.9 kW, the predicted heat flux matches the experimental value less accurately, both qualitatively and quantitatively. This is likely due to the inability of the model to simulate flame lift-off and accurately predict the convective heat transfer in the lower laminar region without utilising a much finer grid.

The results from the present work show that the current model is capable of predicting the general trend of the cavity fire with varying widths. While the model has predicted the heat flux at the laminar region and flame height less accurately. The goal of the research is to develop a model capable of studying the fire dynamics within a cavity in a facade fire configuration. The model requires further development and research, such as mechanical failures in a facade fire which was not modelled in this study. However, the model is suitable for study scenarios where the majority of the flow is turbulent, and the cavity is in a well-ventilated condition. It is important to note that many of the issues found are known issues in the field. However, for the specific scenario at hand, narrow cavity fire, computational modelling is not abundant, and the current work intends also to highlight the complexity of multiphysics modelling of narrow cavity fire.

## Supplementary Information

Below is the link to the electronic supplementary material.

Supplementary file1 (DOCX 522 KB)

## Data Availability

Data is available from the authors upon reasonable request.
