# Supplementary material for: Fire Inside the Cavity of a Non-flammable Facade: Step-by-Step Development of Multiphysics Computer Simulations
Source: Fire Technol. 2024 Dec 24;61(4):2235–63. doi: 10.1007/s10694-024-01680-z (PMC12134032; doi:10.1007/s10694-024-01680-z)
Supplement: Supplementary file 1 — Supplementary file1 (DOCX 522 KB) [file 10694_2024_1680_MOESM1_ESM.docx]

# Appendix

This appendix provides additional figures on the numerical domain used and extra validated simulation results for S1 Fluid, S2 Heat and S3 Buoyancy. These appendixes intend to give a better picture of the numerical setup and provide extra confidence in the model’s capability to predict each physics present in these.

## Turbulence model

The WALE model computes the SGS eddy viscosity, $\nu_{sgs}$ as follows:

|  | $\nu_{sgs}=\left( C_{w}\Delta^{2} \right)\frac{\left( S_{ij}^{d}S_{ij}^{d} \right)^{\frac{3}{2}}}{\left( \tilde{S}_{ij}\tilde{S}_{ij} \right)^{\frac{5}{2}}+\left( S_{ij}^{d}S_{ij}^{d} \right)^{\frac{5}{4}}}$ | (9) |
| --- | --- | --- |

Where C­_w_ is the model constant with a coefficient of C­_w_=0.55 based on [52]. $\Delta$ is the LES filter size, $S_{ij}^{d}$ is the special tensor defined by Nicoud [51], and $\tilde{S}_{ij}$ is the resolved scale strain rate tensor. The SGS turbulent kinetic energy, $k_{sgs}$ and the rate of dissipation of SGS turbulent kinetic energy in WALE is expressed, as shown below:

|  | $k_{sgs}=\left( \frac{\nu_{sgs}}{C_{k}\Delta} \right)^{2}$ | (10) |
| --- | --- | --- |
|  | $\varepsilon_{sgs}=C_{E}\frac{k_{sgs}^{\frac{3}{2}}}{\Delta}$ | (11) |

Where C_k_ and C_E_ are the model constants, where values are based on [52] (C_k_= 0.29 and C_E_=1.048).

However, to ensure the WALE model implemented is suitable for cavity flow, both Smagorinsky and k-equation eddy viscosity models are used in S1 Flow and S2 Heat to compare the differences between the models.

In the Smagorinsky model, $\nu_{sgs}$ is modelled as follows:

|  | $\nu_{sgs}=\left( C_{s}\Delta\right)^{2}\left\vert\tilde{S} \right\vert$ | (12) |
| --- | --- | --- |

Where C­_s_ is the model constant with a range of 0.1 to 0.25 [63]. In the present study, the coefficient used is C­_s_=0.1 based on [53].$\left| \tilde{S} \right|=\sqrt{\tilde{S}_{ij}\tilde{S}_{ij}}$ is the strain rate tensor. To obtain $k_{sgs}$, the Smagorinsky model solves the balance equation shown in (8), with the value of $\varepsilon_{sgs}$ obtained through (5)

|  | $\tilde{S}:B+\frac{C_{E}k_{sgs}^{\frac{3}{2}}}{\Delta}=0$ | (13) |
| --- | --- | --- |

Where “:” is the double inner product operator, $B=\frac{2}{3}k_{sgs}I-2C_{k}\sqrt{k_{sgs}}\Delta\tilde{S}_{D}$ is the SGS stress tensor with $\tilde{S}_{D}$ a the deviatoric component of the strain rate tensor. The model constants C_k_ and C_E_ have a coefficient of 0.05 and 1.048, respectively.

For the k-equation model, the $\nu_{sgs}$ is calculated as:

|  | $\nu_{sgs}=C_{k}\Delta\sqrt{k_{sgs}}$ | (14) |
| --- | --- | --- |

Where C_k_ is 0.05. To obtain the $k_{sgs}$, a transport equation is solved as shown:

|  | $\frac{\partial\left( \bar{\rho}k_{sgs} \right)}{\partial t}+\frac{\partial\left( \bar{\rho}\tilde{u_{i}}k_{sgs} \right)}{\partial x_{i}}-\frac{\partial}{\partial x_{i}}\left( \bar{\rho}\left( \nu+\nu_{sgs} \right)\frac{\partial k_{sgs}}{\partial x_{i}} \right) =-\frac{2}{3}\left( \bar{\rho}k_{sgs}+\bar{\rho}\nu_{sgs}\frac{\partial\tilde{u}_{k}}{\partial x_{k}} \right)\frac{\partial\tilde{u}_{i}}{\partial x_{i}}+2\bar{\rho}\nu_{sgs}\frac{\partial\tilde{u}_{i}}{\partial x_{j}}\frac{\partial\tilde{u}_{j}}{\partial x_{i}}-\bar{\rho}\varepsilon_{sgs}$ | (15) |
| --- | --- | --- |

The closure expression for $\varepsilon_{sgs}$ uses the same expression in (5) with C_E_ =1.048.

## Computational Domain

| 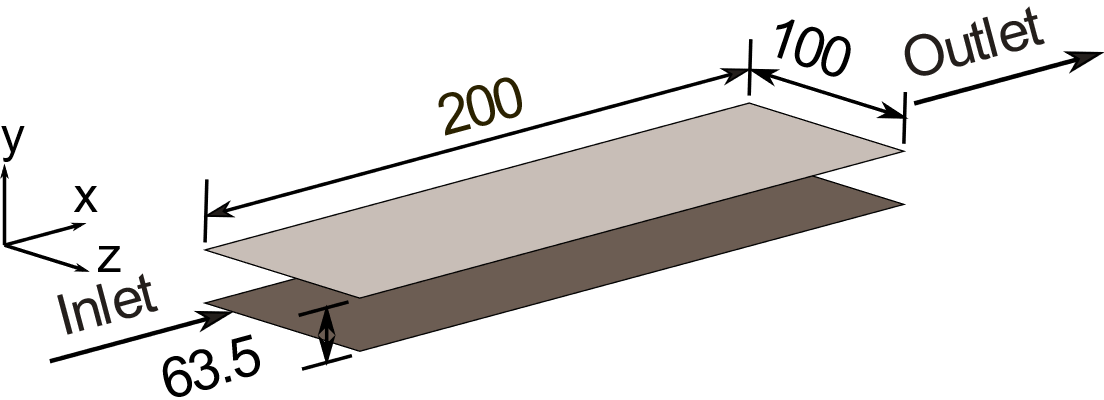 | 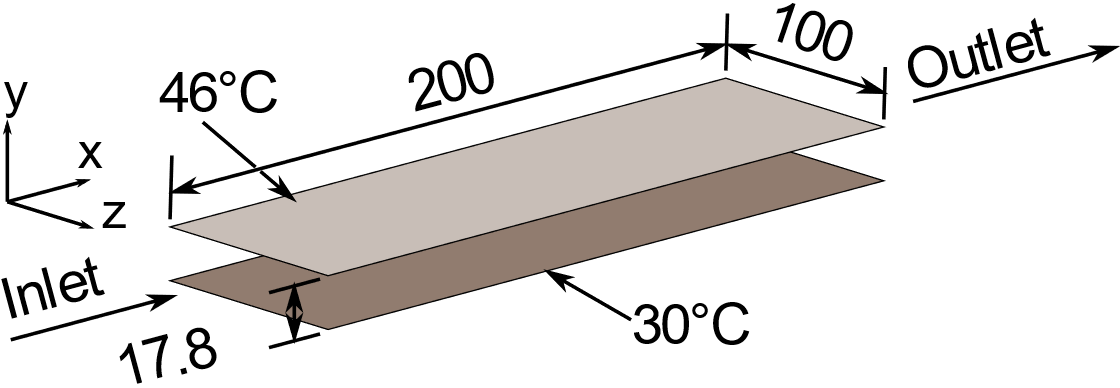 |
| --- | --- |
| Appendix 1 **Numerical domain of S1 Flow. All dimensions are in millimetres (mm).** | Appendix 2 **Numerical domain of S2 Heat. Dimensions are in milimetres (mm).** |


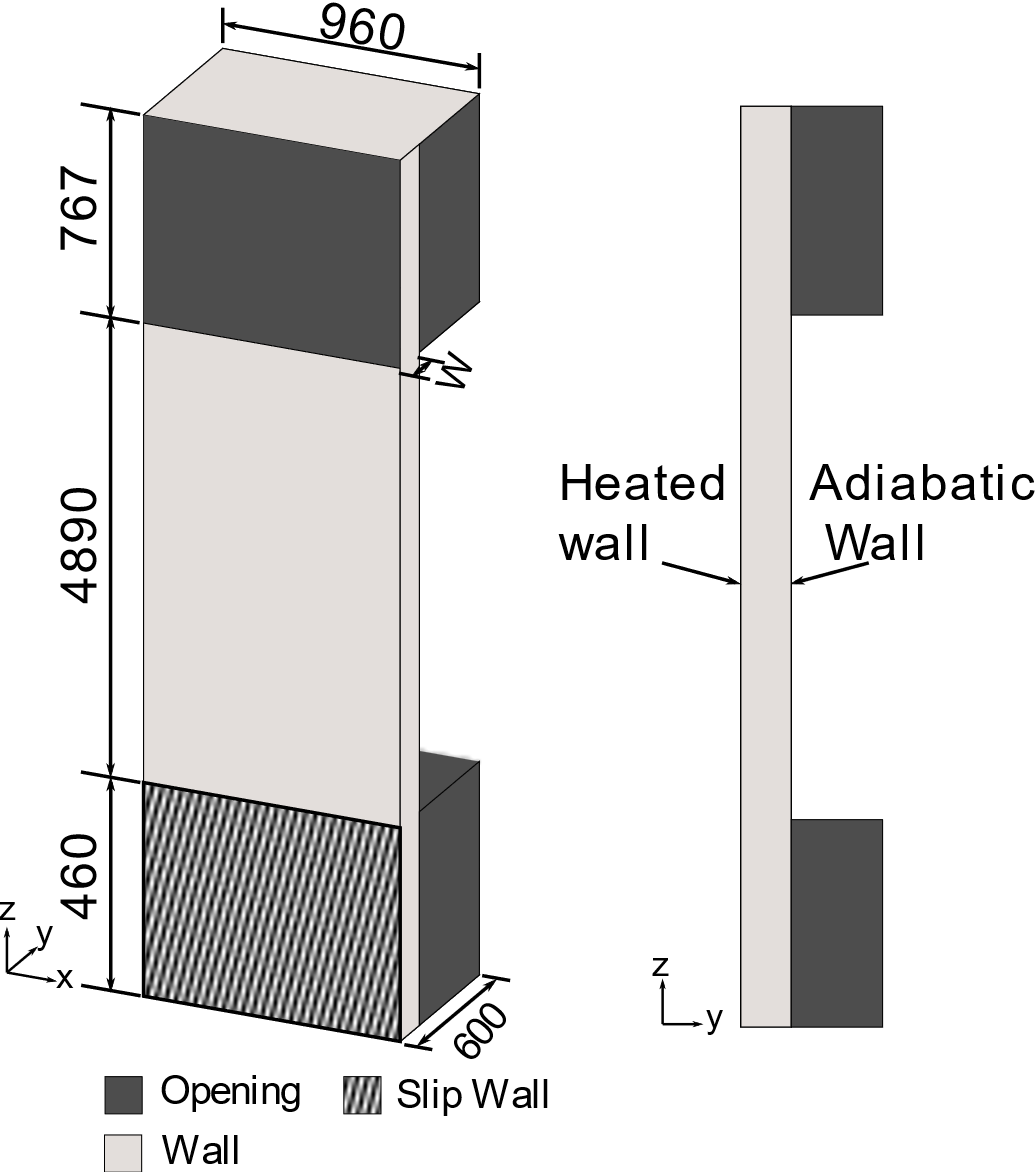


Appendix 3 **Numerical Domain of S3 Buoyancy. Due to symmetry, only one cavity channel was simulated. Dimensions are in millimetres (mm)**

## Results

| 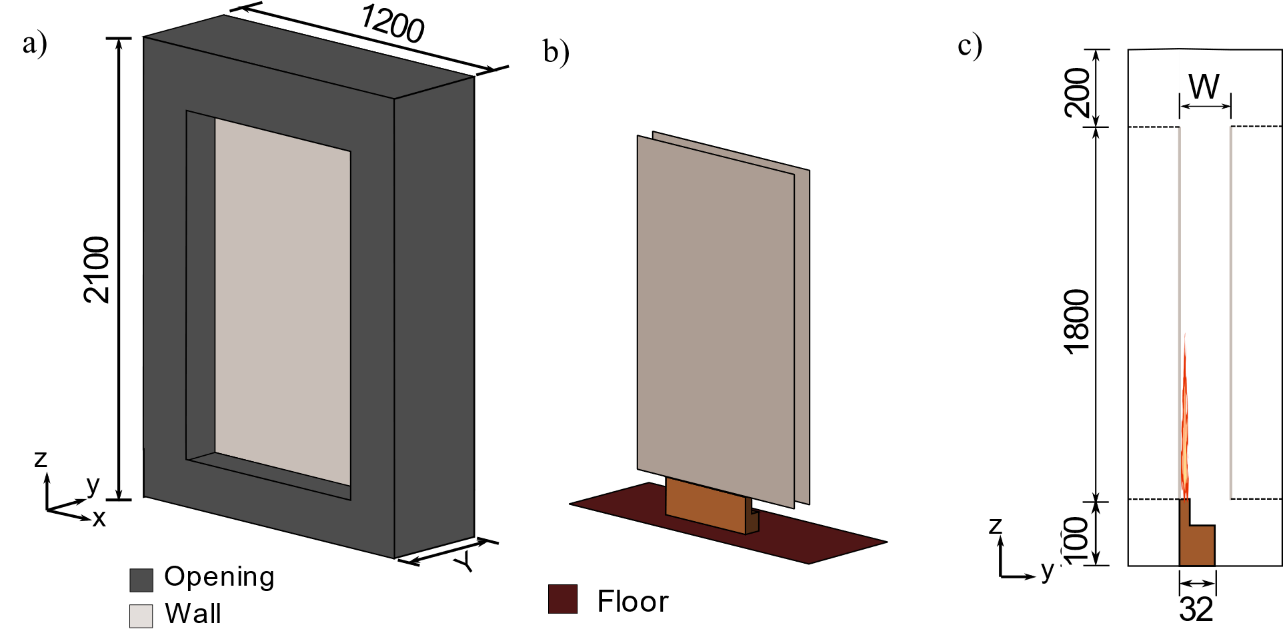 |
| --- |
| Appendix 4 **a)Numerical Domain of S4 Combustion with domain with all boundaries shown. b) Opening was hidden to show both the burner and facade cavity. c) Cross-section of the numerical domain. All dimensions are in millimetres (mm)** |

**Appendix 5 Scenario 2 Heat simulation result compared to experimental for a) variation of temperature with velocity and b)** Wall normal variation of SGS kinetic energy**. Good agreement with the experimental result was found for all turbulence submodel, although WALE model better at predicting the vanishing SGS kinetic energy at near the wall.**

**Appendix 6 Comparison of experimental and predicted Velocity profile for cavity widths of W = 50 mm, 100 mm, and 200 mm at the height of 820 mm, 2655 mm and 3865 mm for q_w_ = 208 W**

## Error analysis

The error analysis between the model and the experiment is obtained by comparing the global features, first-order statistical moments and, if experimental data is available, the second-order statistical moments. These comparisons are made by evaluating the mean relative error and bounded relative error between the experimental data and simulation prediction, as shown in (16) and (17)

|  | $RE^{m}=\frac{1}{N}\sum_{i=1}^{i=N} \left( \frac{M_{i}-E_{i}}{E_{i}} \right)\times100$ | (16) |
| --- | --- | --- |
|  | $RE^{b}=\sum_{i=1}^{i=N} \tanh\left( \frac{M_{i}-E_{i}}{E_{i}} \right)\times100$ | (17) |

Where $RE^{m}$ is the mean relative error, $RE^{b}$ is the bounded relative error, M is the predicted value, and E is the experimental value. Uncertainty of the error is also calculated for experiments that provide information on experimental uncertainty.

$RE^{b}$ was also used to avoid the large relative error when the measured value is close to zero.

**Appendix 7** Mean Relative error and bounded relative error for Scenario 1 over the various measurements. Smagorinksy and K-equation model performs slightly better due to the Van Driest wall function.

|  | Mean velocity,$\bar{\boldsymbol{U}}$ | | Log law velocity, $\frac{\boldsymbol{U}^{\boldsymbol{+}}}{\boldsymbol{U}_{\boldsymbol{\tau}}}$ | | Velocity intensity, $\frac{\sqrt{\bar{\boldsymbol{u}^{\boldsymbol{'2}}}}}{\bar{\boldsymbol{U}}}$ | |
| --- | --- | --- | --- | --- | --- | --- |
|  | $RE^{M}$ | $RE^{b}$ | $RE^{M}$ | $RE^{b}$ | $RE^{M}$ | $RE^{b}$ |
| WALE | 6.4% | 6.3% | 12.4% | 12.3% | 24.8% | 23.2% |
| Smagorinsky | 5.9% | 5.9% | 7.9% | 7.8% | 19.7% | 18.2% |
| K-equation | 5.8% | 5.6% | 7.6% | 7.6% | 21.4% | 19.7% |

**Appendix 8** Mean Relative error and bounded relative error for Scenario 2 over the various measurements. WALE model performs better likely due to better prediction of ν_sgs_ near walls

|  | Mean velocity,$\bar{\boldsymbol{U}}$ | | Temperature, $\boldsymbol{T}$ | | Dimensionless Mean Temperature, $\frac{{\bar{\boldsymbol{T}}}^{\boldsymbol{+}}}{\boldsymbol{Pr}}$ | | Dimensionless Temperature Fluctuation, $\frac{\boldsymbol{\theta}^{\boldsymbol{'+}}}{\boldsymbol{Pr}}$ | |
| --- | --- | --- | --- | --- | --- | --- | --- | --- |
|  | $RE^{M}$ | $RE^{b}$ | $RE^{M}$ | $RE^{b}$ | $RE^{M}$ | $RE^{b}$ | $RE^{M}$ | $RE^{b}$ |
| WALE | 4.6% | 4.6% | 0.6% | 0.6% | 14.7% | 14.5% | 8.8% | 6.7% |
| Smagorinsky | 11.9% | 11.7% | 1.3% | 1.3% | 16.1% | 15.8% | 12.4% | 9.6% |
| K-equation | 8.8% | 8.8% | 0.9% | 0.9% | 15.7% | 15.5% | 14.5% | 11.2% |

**Appendix 9** Mean Relative error and bounded relative error for Scenario 3 over the various measurements. After the addition of buoyancy, the relative error increased drastically.

| Cavity width, mm | Mean velocity,$\bar{\boldsymbol{U}}$ (208 W) | | Temperature, $\boldsymbol{T}$ (104 W) | | Velocity fluctuation, (104W) | | Temperature Fluctuation, $\frac{\sqrt{{\bar{\boldsymbol{T}}}^{\boldsymbol{2}}}}{\boldsymbol{T}}$ (104 W) | |
| --- | --- | --- | --- | --- | --- | --- | --- | --- |
|  | $RE^{M}$ | $RE^{b}$ | $RE^{M}$ | $RE^{b}$ | $RE^{M}$ | $RE^{b}$ | $RE^{M}$ | $RE^{b}$ |
| 50 | 24.6 ± 8% | 23.9 ± 8% | 17.1 ± 10% | 16.9 ± 10% | N/A | N/A | N/A | N/A |
| 100 | 28.4 ± 8% | 27.3 ± 8% | 16.8 ± 10% | 16.6 ± 10% | 40.3% | 34.6% | 23.4% | 22.4% |
| 200 | 49.0 ± 8% | 39.7 ± 8% | 14.0 ± 10% | 13.9 ± 10% | 24.2% | 22.3% | N/A | N/A |
